# Alterations to adipose tissue morphology during inflammatory arthritis is indicative of vasculopathology in DBA/1 mice

**DOI:** 10.1080/21623945.2017.1295174

**Published:** 2017-02-21

**Authors:** Katie Sime, Ernest H. Choy, Anwen S. Williams

**Affiliations:** a Division of Infection and Immunity, Department of Rheumatology, Cardiff University School of Medicine, Cardiff, United Kingdom; b The Cardiff Regional Experimental Arthritis Treatment and Evaluation Centre (CREATE Centre), Cardiff University School of Medicine, Cardiff, United Kingdom

**Keywords:** Adipose tissue, perivascular adipose tissue, inflammatory arthritis, vasculature, macrophage, galectin-3

## Abstract

The physiologic function of adipose tissue is altered by the host's inflammatory response; the implications for maintaining human health and regulating inflammation-associated disease progression are ill defined. However, this cannot be investigated in humans, therefore the use of animal models is required. With the aim to determine morphological and molecular alterations to perivascular and organ-associated adipose tissues during inflammatory arthritis, collagen-induced arthritis (CIA) was established in male DBA/1 mice. Emerging evidence from this study signposts CIA in the DBA/1 mouse as a model that is relevant to study the development and treatment of early cardiovascular pathology associated with inflammatory arthritis. Here, we show global morphological changes in adipose tissue and the thoracic aorta in animals induced with CIA compared with the non-immunized controls. In CIA, we concluded that the increased cell count in PVAT was, at least in part, caused by an ingress and/or expansion of macrophages that had a mixed phenotype. A substantial increase of galectin-3 was expressed in PVAT from mice with CIA. Galectin-3 is elevated in the blood of patients with CVDs, however, it has never before been measured in PVAT in rodents or humans. Here, PVAT-associated galectin-3 is identified as a potential biomarker for detecting early vascular pathology in CIA and a promising candidate for translation to RA.

## Introduction

The prevalence of being overweight and obesity are increasing worldwide, and are also surprising features in patients with rheumatoid arthritis (RA) that is normally associated with hyper-catabolism or rheumatoid cachexia.^1,2^ Increased fat mass is accompanied by decreased lean mass in patients with RA.^3,4^ This aberrant change in fat/lean mass ratio is associated with reduced efficacy of disease modifying anti-rheumatic drugs^5^ and anti-tumor necrosis factor (anti-TNF) therapy.^6^ Adipose tissue is a potent source of cytokines and adipokines including TNF.^7^ By orchestrating systemic inflammation, disease progression and sub-optimal therapeutic responses, the influence of adipose tissue on RA pathology is multifaceted. Altered fat/lean body composition is also linked to hypertension, low high-density lipoprotein levels, insulin resistance and metabolic syndrome in RA.^8^ The role of adipose tissue in modulating RA-associated comorbidities is likely underestimated. Although published data are sparse and contentious, emerging evidence suggests that the proportions and bodily distribution of fat predisposes individuals to diabetes, hypertension and risk for cardiovascular disease. This study, to our knowledge, is the first to describe the morphology of perivascular adipose tissue (PVAT), white adipose tissue (WAT) and brown adipose tissue (BAT) in DBA/1 mice. This strain is widely used to study therapeutic, pathological and immunological responses in the collagen-induced arthritis (CIA) model; the favored surrogate experimental model for RA. Here, CIA was used to distinguish phenotypic changes to adipose tissue during inflammatory arthritis that may well be implicit to identifying modifiable factors for future advancement of disease management strategies.

Adipose tissue is required to fulfil several important physiologic functions that include structural support, lipid storage, thermogenesis and tissue homeostasis.^9^ The adipose organ comprises 2 tissue types: white adipose tissue (WAT) and brown adipose tissue (BAT).^10,11^ The function of adipose tissue largely depends on its location. Perivascular adipose tissue (PVAT) surrounds systemic blood vessels; it is an abundant mixture of WAT and BAT^12^ and secretes adipokines (e.g. adiponectin and leptin), cytokines (e.g., Interleukin-6, Interleukin-8 and TNFα) and gaseous molecules such as reactive oxygen species (ROS) and H_2_O_2_.^13–17^ These soluble factors regulate dilation and constriction responses in the vasculature ^18^ and modulate immune function.^19,20^ The mechanisms that underlie arthritis-associated cardiovascular pathologies may well be linked to PVAT. A notion that is supported by studies in obesity and cardiovascular disease that show PVAT's normal inhibitory action on vascular constriction responses are counteracted by oxidative stress and inflammation in the adipose tissue environment.^18^ Data from human studies translates across several mammalian species (including mouse) and animal models that also report the negative functional impact of adipose tissue associated inflammation upon the vasculature.^21,22^ DBA/1 mice exhibit CIA-associated contractile dysfunction in the thoracic aorta.^23^ This study will determine changes to PVAT morphology during CIA that could heighten risk of developing arthritis-associated cardiovascular pathology and dysfunction.

In patients with diabetes, hypertension and obesity changes to adipose tissue structure are observed namely; adipocyte hypertrophy, increased mass, altered lipid droplet composition and tissue infiltration by macrophages and T cells.^22,24^ WAT is a dense network of white adipocytes containing a single large lipid droplet with very few mitochondria^25,26^ that is characterized by the expression of several protein markers that include the amino acid transporter Asc-1.^27^ BAT, differs in morphology to WAT. The adipocytes in BAT contain multiple lipid droplets and higher numbers of mitochondria.^25,26^ BAT is characterized and differentiated from WAT by its expression of the mitochondrial proton carrier uncoupling protein 1 (UCP-1) and a member of the proton-coupled amino acid transport (PAT) family, PAT2.^28,29^ PVAT is complex adipose tissue depot termed ‘beige’ because it is composed of brown-like white adipocytes; effectively a of mixture of WAT and BAT.^30^ Like BAT, beige adipocytes express UCP-1 but contain fewer and slightly larger lipid droplets and an intermediate mitochondrial density that lies between WAT and BAT.^25,31^ Beige adipocytes are a distinct cell population and their primary function is reported to be thermogenesis. Here, and to our knowledge for the first time, we describe PVAT, WAT and BAT changes in morphology caused by CIA and measure consequential changes in WAT, BAT and macrophage-associated molecular signals in this model of inflammatory arthritis. This novel approach constitutes a first step toward the identification of early disease activity markers for therapy, diagnosis or prognosis of organ-associated co-morbidities for RA. Our data identify PVAT-associated galectin-3 and M2 macrophages as novel targets to prevent, treat or detect vascular dysfunction at the earliest stages of inflammatory arthritis. This concept, if applicable to humans, has major clinical implications.

## Materials and Methods

### Animals and CIA induction

All animal care and experimental procedures complied with the United Kingdom Animals (Scientific Procedures) Act 1986 in accordance with project license 30/2928 and approved by Local Research Ethics Committee. CIA was initiated in 8 week-old male DBA/1 mice (n = 8) (Harlan) as described previously.^23,32,33^ Mice were injected intradermally under anesthetic (Isoflurane + O_2_) with an emulsion consisting of type II chicken sternal collagen (1mg/ml)(Sigma Aldrich, C9301–5MG) and Complete Freund's Adjuvant (2.5mg/ml) on Day 0 and received a second identical immunization on Day 21. Thereafter, mice were monitored daily by recording hind paw diameters and scoring macroscopic poly-articular features of CIA in each paw (paw score: 0 = no arthritis, 1 = mild/moderate erythema and swelling, 2 = severe swelling covering entire paw, 3 = 3 joints affected by arthritis, 4 = all joints affected by arthritis, 5 = Deformed paw/ankyloses). Individual paw scores were combined to give a comparative daily clinical score for each mouse. Age-matched (n = 4) naïve, non-immunized, male mice were used as controls (no arthritis). Mice were killed by inhalation of rising CO_2_ concentration, followed by cardiac puncture. Mice were fed standard rodent chow and had water ad libitum.

### Histological assessment of arthritis

Hind paws comprising joints of the ankle and foot were harvested at experiment end, fixed in 1% neutral buffered formalin solution, decalcified in ethylenediaminetetracetic acid, processed through to paraffin wax, embedded and cut into sections. Tissue sections (7μm) were cut, mounted onto glass slides and stained using hematoxylin and eosin (H&E). Images were acquired using ZEISS Observer Z.1 microscope and analyzed using Zen Pro 2 computer package (ZEISS, UK). Joint tissue sections were analyzed by 2 blinded independent observers as described previously.^32,34^ Arthritis-associated histological features were graded as follows: cellular infiltration of sub-synovial tissue (0–5), synovial hyperplasia and pannus formation (0–3), cell exudate in joint space (0–3) and bone erosion (0–3). The total score of all parameters gave the arthritis index (AI; mean ± SEM reported).

### Histological analysis of adipose tissues

Thoracic aorta (PVAT intact), abdominal aorta (PVAT intact), WAT (renal and gonadal) and BAT (inter-scapular) were harvested, fixed in 70% v/v ethanol and processed through to paraffin wax blocks. Adipose tissue sections were stained with H&E, visualized using light microscopy and analyzed using Zen Pro 2 computer package (ZEISS, UK). Areas of interest were selected, covering 70% of each tissue type per section respectively (PVAT, blood vessel wall, WAT and BAT) and the cell counter Plugin (Image J) was used to calculate average cells per mm^2^. The thickness (lumen to adventitia) of the blood vessel wall was measured across 12 equidistant regions that covered the entire vessel. Subtle fatty changes were expressed numerically by calculating pixels contained in lipid droplets versus total per region of interest (expressed as percentage) using Adobe Photoshop Version6 in each adipose tissue section (termed vacuolarity). All data reported as mean ± SEM.

### Measurements of cytokine plasma concentrations using LEGENDplex assay

Plasma concentrations of cytokines TNF-α, IFN-γ, IL-6, IL-10, IL-4, IL-13, IL-2, IL-5, IL-9, IL-17, IL-21 and IL-22 were determined following manufacturing instructions of the LEGENDplex assay (BioLegend, UK).

### Quantitative real-time polymerase chain reaction (qPCR)

Cellular RNA from freshly excised PVAT (thoracic and abdominal), WAT (renal and gonadal) and BAT (inter-scapular) was stabilized and protected using RNAlater® solution (ThermoFisher Scientific, AM7020). Tissues were homogenized individually. RNA was isolated using chloroform and isopropanol. A high capacity RNA-cDNA kit (Applied Biosystems, 4387406) was used to convert the RNA into cDNA. Samples were heated on a thermocycler (Applied Biosystems) at 37°C for 60mins before heating to 95°C to stop the reaction. The 96 well Fast SYBR Green (Life Technologies, 4472908) Δ-ΔCᴛ qPCR method (ViiA7 qPCR machine; Applied Biosystems) was used to quantify gene expression. The following primers were used: Asc-1 (FP: ACAGGCCAGGATCTAAGGTG, RP: CCTCGTGGGGTCTCAACATA), PAT2 (FP: CCTTCCTGAGAGTGCCAAGA, RP: TGTCTGGAACCCGGTTATGC), galectin-3 (FP: ACCCAACGCAAACAGGATTG, RP: TGTCCTGCTTCGTGTTACAC), iNOS (FP: TATTTTCAGGGCTTGCGTGG, RP: CCACCTCTCCTGGCTTGATG), CD11c (FP: ACGCTTACCTGGGTTACTCC, RP: AAGATGACAACCTTCCCCGT), Arg1 (FP: ACAAGACAGGGCTCCTTTCA, RP: TGCCGTGTTCACAGTACTCT) and CD206 (FP: GTTCAGCTATTGGACGCGAG, RP: AGTTGCCGTCTGAACTGAGA). The normalization control was β-actin (FP: TGGCACCACACCTTCTACAA, RP: AGGTCTCAAACATGATCTGGGT). Results were presented as the average change in expression (CIA vs. naïve) in DBA/1 wild-type mice.

### Statistical analysis

Results are presented as mean ± SEM values. Differences between 2 groups were evaluated by the unpaired Student's t test if they passed the Kolmogorov–Smirnov normality test or otherwise by the non-parametric Mann–Whitney test as indicated. Differences between more than 2 groups were evaluated by Kruskal–Wallis test with Dunn's multiple comparison test. P values of ≤ 0.05 denote significant changes. Graphical plots and statistical analyses were performed using GraphPad Prism v5.0.

## Results

### Robust polyarthritis was observed in all immunized DBA/1 mice

**Figure 1.**
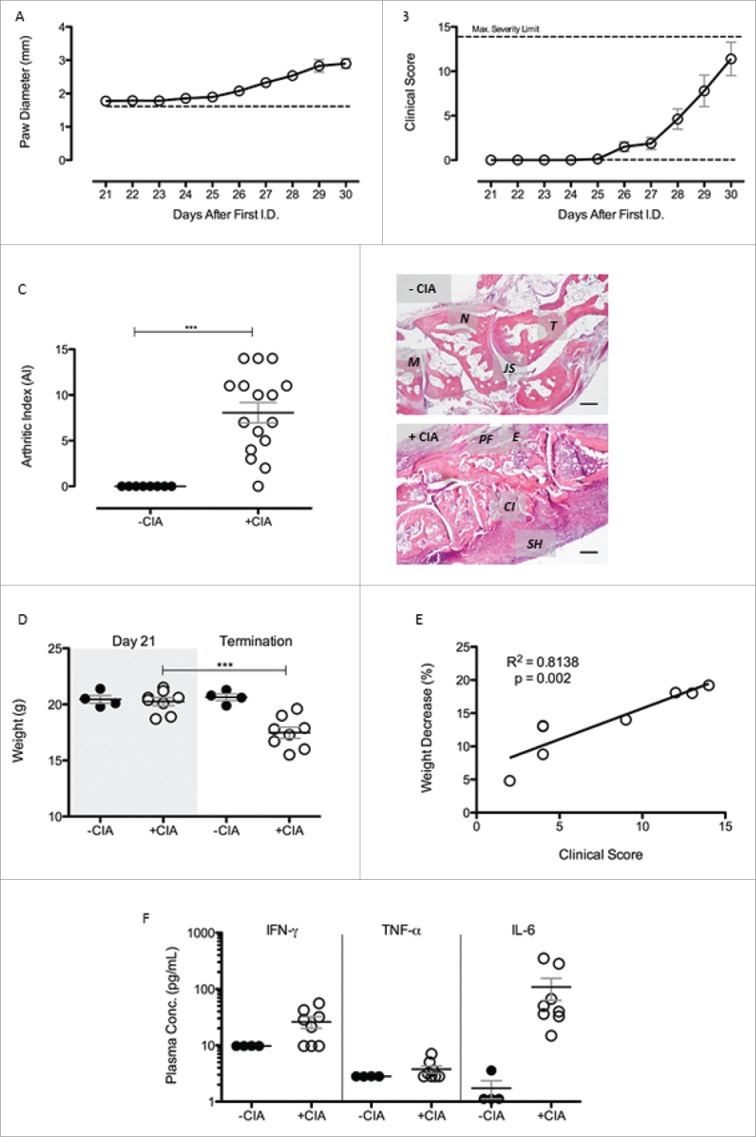
CIA was successful induced in male DBA/1 mice (A) Paw diameter was measured throughout the progression of arthritis in +CIA mice (○). –CIA (dashed line) is equal to average paw diameter scores of non-immunized control mice at termination. (B) Clinical Score was calculated throughout progression of arthritis and with a maximum severity limit of 14. –CIA (n = 4), +CIA (n = 8). (C) Severity of arthritis of –CIA (n = 8) and +CIA (n = 16) hind paws was histologically assessed through calculation of arthritic index (AI). Representative –CIA paw AI = 0: cellular infiltration (0–5), synovial hyperplasia (0–3, bone erosion (0–3) and cellular exudate (0–3). Representative +CIA paw AI = 10: cellular infiltration (4–5), synovial hyperplasia (2–3, bone erosion (2–3) and cellular exudate (2–3). *M*: 1^st^ metatarsal, *N*: navicular, *T*: talus, *JS*: joint space, *PF*: pannus formation, *E*: bone erosion, *CI*: cellular infiltrate. *SH*: synovial hyperplasia. Scale bar = 200μm (D) Weight was monitored daily during arthritis progression in –CIA (n = 4) and +CIA (n = 8) mice with an adverse severity limit of 20% weight loss. (E) Correlation between percentage weight decrease and clinical score in +CIA mice (n = 8). (F) Plasma concentration of pro-inflammatory cytokines were measured to assess the systemic inflammatory nature of the CIA model. Data expressed mean ± SEM. *** = p < 0.001.

Animal models were used to discover new therapeutic targets that are currently under investigation for limiting the complex pathogenic responses instigated by adipose tissues.^35–37^ We reasoned that CIA was the most appropriate model to study the pathophysiology of adipose tissue-associated extracellular matrix remodeling during inflammatory arthritis and to unmask potential targets for modulating arthritis-associated cardiovascular disease. We observed 100% incidence of CIA in mice immunized against collagen II and a robust inflammatory response characterized by extensive polyarthritis affecting all paws. Paw diameter measurements were used to gauge joint swelling in the hind paws (Day 21 onwards). Values increased steadily over the time course of the experiment (CIA vs. naïve, Fig. 1A) reaching their maximum at termination (p < 0.001; 2.7 ± 0.1 vs. 1.89 ± 0.02). Clinical score measured macroscopic disease activity and showed CIA progression over time (Fig. 1B), in agreement with published data.^23,32,33,38^ Histological assessment of hind paws from both naïve and CIA mice (Fig. 1C) revealed substantial inflammatory and degenerative pathology that was evidenced by an AI of 8.1 ± 1.1 (mean ± SEM; p < 0.001) compared with the non-immunised controls (AI = 0). During CIA progression, weight decreased from 20.3 ± 0.4 g on Day 21 to 17.5 ± 0.5 g at termination in arthritic mice (p < 0.001), whereas no weight decrease was seen in non-immunized control mice (Fig. 1D). Percentage weight lost over this period had a positive correlation with clinical score (p < 0.01) (Fig. 1E). Plasma concentrations of the pro-inflammatory cytokines tumor necrosis factor-α (TNF-α), interferon-γ (IFN-γ) and interleukin-6 (IL-6) were not significantly different in CIA mice compared with the non-CIA controls (Day 30) (Fig. 1F). Levels of anti-inflammatory cytokines (e.g., IL-10, IL-4 and IL-13) and other T-cell associated cytokines (e.g., IL-2, IL-5, IL-9, IL-17, IL-21 and IL-22) were below detection limit of the LEGENDplex assay in both groups (data not shown).

### Pathological changes in thoracic PVAT and vessel wall of aorta accompany CIA

**Figure 2.**
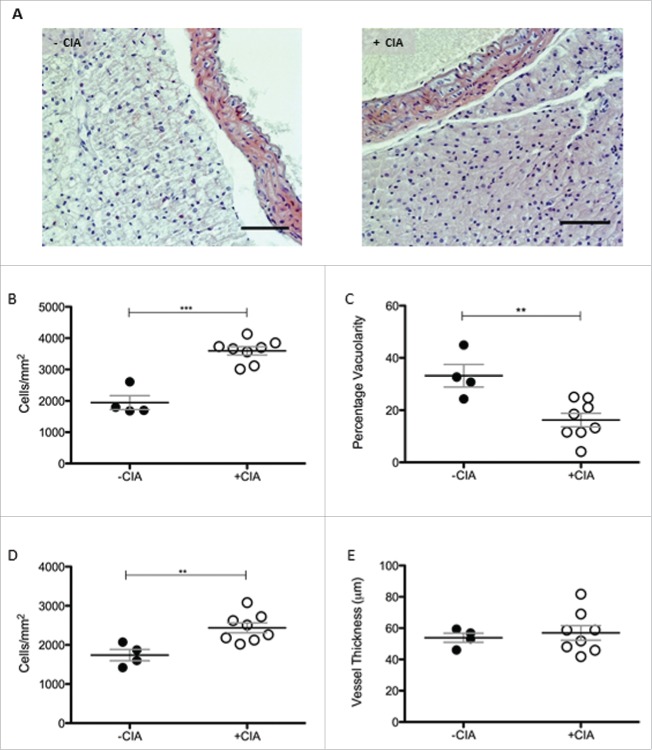
Morphological characterization of thoracic PVAT in DBA/1 naïve and CIA mice (A) Representative images of –CIA and CIA thoracic aorta + PVAT show differences in morphology between the non-arthritic and arthritic mice. Scale bar = 100μm. (B) Total cell number and (C) vacuolarity was analyzed in thoracic PVAT. In the thoracic aorta, (D) total cell number and (E) vessel thickness were measured. –CIA (n = 4), +CIA (n = 8). Data expressed as mean ± SEM. *** = p < 0.001, ** = p < 0.01.

The appearance of thoracic PVAT in naïve mice (Fig. 2A) was reminiscent of descriptors from the published literature namely, a network of adipocytes with large lipid-containing vacuoles and resident immune cells.^26^ CIA created an inflammatory environment (Fig. 2A) in PVAT. Cell number was increased 1.8-fold (p < 0.001; 3594 ± 131 vs. 1948 ± 222 cells/mm^2^) by CIA compared with non-immunized naïve control mice (Fig. 2B). The increased cell density was accompanied by the significant reduction in vacuolarity (p < 0.01) in thoracic PVAT (Fig. 2B). Leukocyte infiltration in the arterial intima sustains chronic inflammation during atherogenesis. Cell number in the vessel wall of thoracic aorta of mice was increased 1.4-fold by CIA compared with mice without arthritis (p < 0.01, 2440 ± 126.8 vs. 1741 ± 144 cells/mm^2^, Fig. 2C). Carotid intima-media thickness is an established surrogate marker of atherosclerosis; this measurement may not reflect whole arterial changes that occur in cardiovascular pathologies like hypertension.^39^ CIA did not thicken the wall of the thoracic aorta (Fig. 2C).

### Morphological changes to PVAT were not unique to the thoracic adipose tissue depot

**Figure 3.**
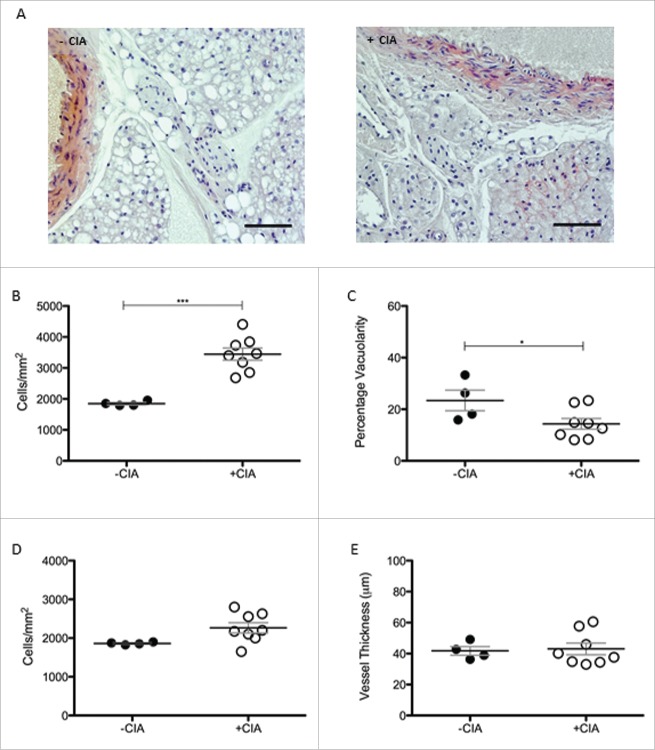
Morphological characterization of abdominal PVAT in DBA/1 naïve and CIA mice (A) Morphological differences in abdominal PVAT between naïve and CIA mice shown in representative images. Scale bar = 100μm. (B) Abdominal PVAT total cell number and (C) vacuolarity were determined in –CIA and +CIA mice. (D) Total cell number and (E) vessel thickness were measured in the abdominal aorta. –CIA (n = 4), +CIA (n = 8). Data expressed as mean ± SEM. *** = p < 0.001, * = p < 0.05.

Tissue remodeling associated with cardiovascular disease and aging is not uniform along the length of the aorta.^40^ The abdominal aorta is relatively stiffer than more proximal sites.^41,42^ In CIA, regional differences were determined by analyzing phenotypic changes to PVAT in the abdominal cavity. Cell counts were significantly (1.9-fold) higher (p < 0.001) in CIA mice vs. the non-arthritic controls (3445 ± 197 vs. 1849 ± 38.1 cells/mm^2^; Fig. 3A, B). A significant decrease (p < 0.05) in the vacuolarity was also observed (Fig. 3B). These morphological changes were comparable with those of the thoracic aorta. In contrast to the thoracic aorta, there was no detectable cell ingress into the wall of the abdominal aorta (Fig. 3C) nor was there any difference in the thickness of the vessel wall (Fig. 3C) during CIA. The abdominal aorta was marginally narrower than the thoracic aorta.

### Morphology of systemic BAT depot but not WAT comparable to PVAT during CIA

**Figure 4.**
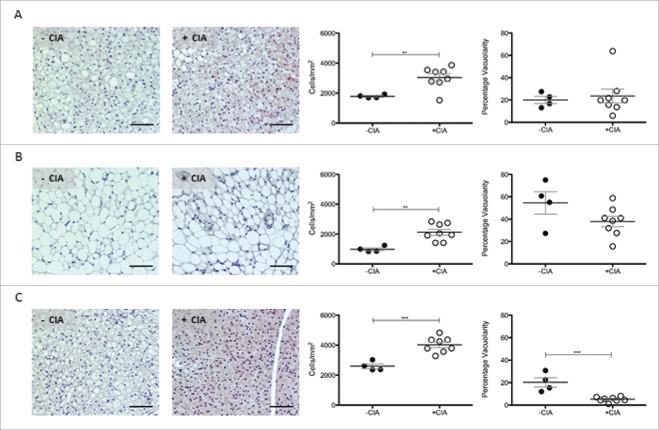
Characterization of renal WAT, gonadal WAT and inter-scapular BAT in DBA/1 naïve and CIA mice. Representative images, total cell number and vacuolarity was analyzed in non-vasculature associated sites in –CIA and +CIA mice: (A-C) Renal WAT (D-F) Gonadal WAT (G-I) Inter-scapular BAT. –CIA (n = 4), +CIA (n = 8). Data expressed as mean ± SEM. *** = p < 0.001, ** = p < 0.01.

In rodents, PVAT that surrounds the abdominal aorta is WAT-like whereas PVAT from the thoracic aorta is quite different and expresses some BAT genes.^11^ Next, comparative histological analyses were determined in adipose tissue sites distant from the aorta. Cell counts in renal-WAT (p < 0.01; 3030 ± 256 vs. 1783 ± 53) and gonadal-WAT (p < 0.01; 2120 ± 201 vs. 978 ± 94.5) were significantly increased in mice with CIA vs. non-arthritic controls. Contrary to our observations in PVAT, there was no significant change in WAT vacuolarity by CIA (Fig. 4A, B). In inter-scapular BAT, cell ingress was also significantly (p < 0.001) increased in CIA vs. naïve mice (4023 ± 178 vs. 2593 ± 154) and it was accompanied by reduced vacuolarity (p < 0.001; Fig. 4C).

### Atherogenesis-associated tissue remodeling markers induced by CIA in PVAT

**Figure 5.**
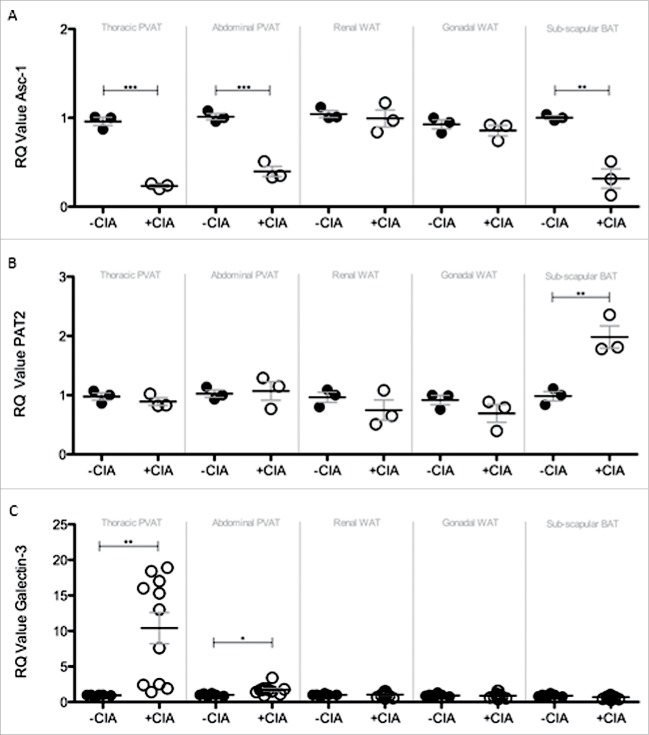
Compositional and tissue remodelling analysis of PVAT, WAT and BAT in –CIA and +CIA DBA/1 mice. The expression of (A) WAT marker ASc-1, (B) BAT marker PAT2 and (C) tissue remodeling marker Galectin-3 were analyzed through qPCR in –CIA (n = 3 (Asc-1 and PAT2), n = 6 (Galectin-3)) and +CIA mice (n = 3 (Asc-1 and PAT2), n = 11 (Galectin-3)). Data is expressed as fold change relative to –CIA mice, mean ± SEM. Housekeeping gene = β-actin. *** = p<0.001, ** = p<0.01, * = p<0.05.

**Figure 6.**
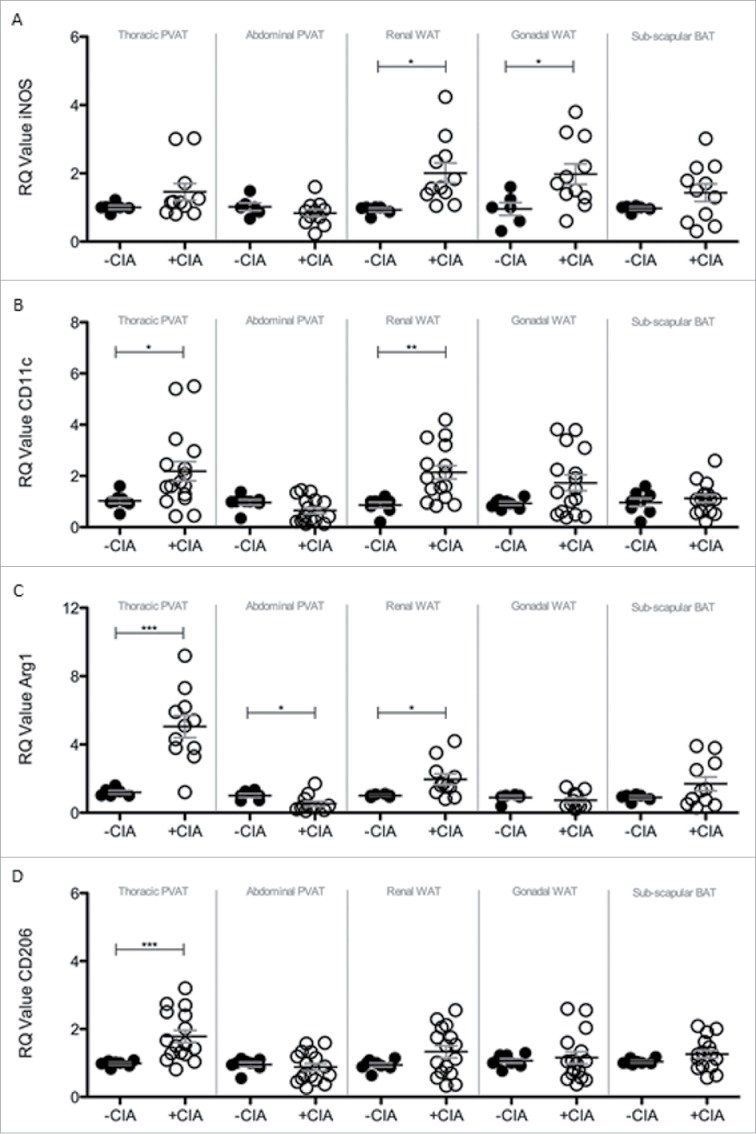
Analysis of M1 and M2 markers in PVAT, WAT and BAT sites of –CIA and +CIA DBA/1 mice. The expression of M1 markers: (A) iNOS and (B) CD11c, and M2 markers: (C) Arg1 and (D) CD206 were analyzed through qPCR in –CIA (n = 6 (iNOS and Arg1), n = 8 (CD11c and CD206)) and +CIA (n = 11 (iNOS and Arg1), n = 16 (CD11c and CD206)). Data is expressed as fold change relative to –CIA mice, mean ± SEM. Housekeeping gene = β-actin. *** = p < 0.001, ** = p < 0.01, * = p < 0.05.

Brown adipocytes reside in BAT as well as WAT depots and in mice the activation of BAT lowers atherogenic-lipoprotein levels, protects against atherosclerosis development^43^ and confers beneficial effects on adiposity, insulin resistance and hyperlipidaemia.^44^ Since systemic inflammation leads to marked adipose tissue remodeling we questioned whether CIA altered the so-called ‘brown-ness’ of PVAT. Expression of the WAT marker Asc-1 was significantly decreased by CIA in thoracic PVAT, abdominal PVAT and inter-scapular BAT vs. naïve mice (Fig. 5A). CIA augmented the expression of the BAT marker PAT2 in inter-scapular adipose tissue (Fig. 5B). PVAT-associated PAT2 was unaffected by CIA. CIA did not alter renal and gonadal WAT expression of Asc-1 or PAT2. Modifications to adipose tissue architecture are not limited to cell ingress or proliferation and adipocyte hypertrophy, hyperplasia and differentiation but include extracellular matrix (ECM) remodeling. Next galectin-3 (Gal-3) was measured as a global marker of fibrosis, angiogenesis, inflammation and atherogenesis that is associated with cardiovascular risk.^45–47^ Gal-3 was significantly increased by CIA in PVAT but more so in tissue from the thoracic vs. abdominal aorta. Gal-3 expression by WAT and BAT was comparable in CIA and naïve mice (Fig. 5C). Adipose tissue contains a heterogeneous array of cells including pre-adipocytes, adipocytes and macrophages (resident and inflammatory) that orchestrate ECM remodeling. We finally asked whether CIA altered the phenotype of adipose tissue-associated macrophages. Overall, CIA exerted fluctuating increases of M1 profiling genes (iNOS and CD11c) across the adipose tissues tested, except for abdominal PVAT and BAT where no change was observed (Fig. 6A, B). CIA caused a profound increase in M2 profiling genes (Arg1 and CD206) in thoracic PVAT but not in other adipose tissue sites. A subtle but significant change in Arg-1 was noted in abdominal PVAT and renal WAT (Fig. 6C, D).

## Discussion

RA is a strong risk factor for the development of cardiovascular disease (CVD), however, the pathophysiological mechanisms of arterial complications in RA remain to be fully elucidated. Identifying early markers of vascular damage identifies a critical step toward the development of preventative strategies against cardiovascular complications in patients with RA. In obesity, dysfunctional and inflammatory adipose tissue impacts upon the cardiovascular system and contributes to CVD. It is unclear whether this is also the case in RA or indeed if the fat accumulation around the vasculature has a role in the pathogenesis of RA-associated vascular dysfunction. These questions are challenging, if not impossible, to resolve in human subjects due to the heterogeneous clinical presentation of RA patients with CVD and the limited sensitivity of conventional diagnostic for the early detection of developing vascular pathology. This article reveals mechanisms that link CIA and PVAT-associated inflammation that could well initiate vascular dysfunction and CVD during inflammatory arthritis in humans.

PVAT is in intimate contact with large, medium and small diameter arterial beds in several tissues, controls vascular function and remodeling and has unique features that impact vascular biology. In CIA, PVAT was distinguished from other types of adipose tissue by inflammatory and morphological changes that were, to a large extent, independent of the visceral adipose tissue and BAT. Increasing epidemiological and clinical evidence demonstrate that perturbations in adipose tissue quality modulate cardio-metabolic disease. This study justifies the application of CIA model, in DBA/1 mice at least, to study PVAT function during inflammatory arthritis and for the discovery and development of preventive strategies, novel diagnostic tools and new targets for therapy for combatting arthritis-associated cardiovascular disease.

We showed that mice with CIA exhibited PVAT, WAT and BAT morphology that was different from naïve, non-immunized controls. The different origins of the progenitor cells from which each fat depot develops may explain the divergent tissue characteristics triggered by CIA.^48,49^ To our knowledge, this is the first report that documents the profound effect of CIA on adipose tissue composition most particularly with respect to the universal increase in the cell density across all the adipose tissue sites and the striking consistency of the change that ranged from 1.6 to 2.2-fold for inter-scapular BAT and gonadal WAT respectively. These changes reflect the systemic inflammatory nature of this arthritis model. However, there may be several additional reasons for the increase in cell number observed in CIA. Fat consists mostly of adipocytes but also pre-adipocytes, endothelial cells, fibroblasts, mesenchymal cells, macrophages and other leukocytes. Therefore adipocyte hyperplasia, expansion of the resident macrophage population and/or adipose tissue infiltration by leukocytes may also modify the architecture of adipose tissue during CIA. This type of hyper-cellular phenotype was previously assigned to region-specific alterations in adipose tissue function, metabolic profile and inflammatory milieu in both animal models and clinical studies related to obesity.^35^^,^^50–53^ Indeed in obesity and type 2 diabetes, diseases also characterized by a state of chronic inflammation, the expansion of metabolically-active adipose tissues lead to local and systemic inflammation, insulin resistance and alterations in lipids, blood pressure, coagulation and fibrinolysis all of which cause endothelial dysfunction, atherosclerosis and cardiovascular disease.^54,55^ Although CIA-associated changes in WAT and/or BAT function were not tested and are as yet undetermined; we recently reported aberrant PVAT-dependent aortic constriction responses in CIA vs. naïve age-matched DBA/1 mice.^56^ Control of peripheral resistance through contraction and relaxation of constituent vascular smooth muscle is a critical function of muscular arteries and arterioles in vivo. PVAT is anatomically co-localized with atherosclerotic lesions in humans (correlating with plaque burden and vascular calcification). The emerging evidence from our studies signpost CIA in the DBA/1 mouse as a model that is relevant to study the development and treatment of early cardiovascular pathology associated with inflammatory arthritis.^23,56^

Anatomical changes to the aorta by CIA were localized to the thoracic compartment where cell counts were increased in the vessel wall. Leukocytes are normally present in the adventitia of the non-diseased artery wall, however, numbers increase as atherosclerotic plaques develop.^57^ There are structural and functional differences between the thoracic and abdominal aorta that are inherent and that also develop during cardiovascular disease and with aging.^58^ Changes to the anatomy of the thoracic and more distal sections of the aorta by CIA could help identify sites susceptible to calcification and wall stiffening during inflammatory arthritis and unmask underlying mechanisms. CIA caused increased expression of macrophage-associated markers, primarily pro-inflammatory M1 phenotype, in both gonadal and renal visceral WAT depots but not in the inter-scapular BAT. Data from the Framingham Heart Study and human tissue specimens found significantly greater association between vascular inflammation, cardiometabolic risk profiles and atherogenic gene expression in visceral vs. subcutaneous fat.^51^^,^^59–62^ Together these findings evidence of the value studying multiple adipose tissue depots in the CIA model for basic research in cardiology.

PVAT was given critical consideration in the CIA model as it was thought to be the major fat depot for identifying changes in vascular pathology and one that would be most useful for predicting early CVD during inflammatory arthritis. In humans, PVAT is anatomically co-localized with atherosclerotic lesions (correlating with plaque burden and vascular calcification) and the degree of PVAT inflammation correlates with cardiovascular disease.^63^ In CIA, we concluded that the increased cell count in PVAT was, at least in part, caused by an ingress and/or expansion of macrophages that had a mixed phenotype. The selected markers were not exhaustive but sufficient for distinguishing classically activated or M1 type (CD11c and iNOS) from alternatively activated or M2 type (Arg1 and CD206); all except iNOS were significantly elevated in PVAT by CIA. Recent studies of an experimental model of endotoxin shock showed that iNOS deficiency resulted in more severe inflammation with an enhanced M1 macrophage activation phenotype and that iNOS expressed by M1 macrophages reduced M1 macrophage differentiation.^64^ Our data reveals the absence of an important negative regulatory pathway during CIA. The apparent inability of PVAT-associated macrophages to increase iNOS expression during arthritis may be an important early trigger for development of CVD and a mechanism that warrants further investigation in future studies. Whereas M1 macrophages are pro-inflammatory, M2 macrophages are associated with responses to anti-inflammatory reactions, tissue remodeling and fibrosis. CIA-associated tissue remodeling in PVAT was first evidenced by a substantial reduction in the expression of the WAT marker Asc-1. The inhibitory effect of inflammation on adipogenesis is relatively well characterized; pro-inflammatory macrophages are considered to be the main effectors.^65^ PAT2 was unaltered by CIA, this result is contrary to reported impairment of brown adipogenesis by macrophages.^66^ This may simply be explained by the distinct phenotype of PVAT vs. adipose tissues from other anatomic locations and, the added complexity, that in rodents and perhaps humans it comprises both BAT and WAT.^12,67,68^ Never-the-less a consensus is emerging that PVAT is a cause of cardiovascular disease.^69^ PAT2 and Asc-1 are selective markers for brown/beige and white adipocytes in human tissue and may be important factors in determining how PVAT influences focal vascular pathophysiology in rodent models and in humans.

Finally, a substantial increase of galectin-3 expression by CIA was measured in thoracic PVAT compared against abdominal PVAT, WAT and BAT depots. Galectin-3 levels are elevated in the blood of patients with CVDs, however, it has never before been measured in PVAT in rodents or humans. Galectin-3 concentrations in the circulation are associated with incident heart failure, atrial fibrillation, atherosclerosis and mortality.^47,70,71^ The clinical utility of measuring galectin-3 for CVD prediction has not been tested in blood or indeed in PVAT. Here, PVAT-associated galectin-3 is identified as a good biomarker for detecting early vascular pathology in CIA and a promising candidate for translation to RA; an idea underpinned by the knowledge of its critical role in, macrophage chemotaxis, phagocytosis, neutrophil extravasation, oxidative stress, apoptosis, and angiogenesis that in turn trigger cardiometabolic traits such as adiposity, insulin resistance, and hyperglycemia.

In conclusion, circulating galectin-3 concentrations are associated with the clinical outcomes in patients with CVD and RA.47,70–72 This study identifies CIA as a model to assess the clinical utility of galectin-3 determination for CVD prediction in patients with RA. The development of therapeutic agents that target galectin-3 may be important to prevent or halt RA-associated CVD. Here we evidence CIA in DBA/1 mice as a relevant, justifiable in vivo model for conducting pre-clinical assessments of new innovative therapies for the treatment of RA-associated CVD.

## Abbreviations

AI: arthritis index
BAT: brown adipose tissue
CFA: complete Freund's adjuvant
CIA: collagen-induced arthritis
CVD: cardiovascular disease
H&E: hematoxylin and eosin
PVAT: perivascular adipose tissue
qPCR: quantitative real-time polymerase chain reaction
RA: rheumatoid arthritis
ROS: reactive oxygen species
TNFα: tumor necrosis factor ɑ
WAT: white adipose tissue

## Disclosure of potential conflicts of interest

No potential conflicts of interest were disclosed.
